# Coercive Field Control in Epitaxial Ferroelectric Hf_0.5_Zr_0.5_O_2_ Thin Films by Nanostructure Engineering

**DOI:** 10.1021/acsami.4c21787

**Published:** 2025-04-15

**Authors:** Ji Soo Kim, Nives Strkalj, Alexandre Silva, Veniero Lenzi, Luis Marques, Megan O. Hill, Ziyi Yuan, Yi-Xuan Liu, Maximilian T. Becker, Simon M. Fairclough, Caterina Ducati, Yizhi Zhang, Jianan Shen, Zedong Hu, Hongyi Dou, Haiyan Wang, José P. B. Silva, Judith L. MacManus-Driscoll

**Affiliations:** † Department of Materials Science & Metallurgy, University of Cambridge, 27 Charles Babbage Road, Cambridge CB3 0FS, United Kingdom; ‡ Physics Center of Minho and Porto Universities (CF-UM-UP), University of Minho, Campus de Gualtar, 4710-057 Braga, Portugal; § Laboratory of Physics for Materials and Emergent Technologies, LapMET, University of Minho, 4710-057 Braga, Portugal; ∥ CICECO - Aveiro Institute of Materials, Department of Chemistry, University of Aveiro, 3810-193 Aveiro, Portugal; ⊥ School of Materials Engineering, Purdue University, West Lafayette, Indiana 47907, United States; # Elmore Family School of Electrical and Computer Engineering, Purdue University, West Lafayette, Indiana 47907, United States

**Keywords:** Ferroelectrics, Hafnia, Epitaxy, Pulsed Laser Deposition

## Abstract

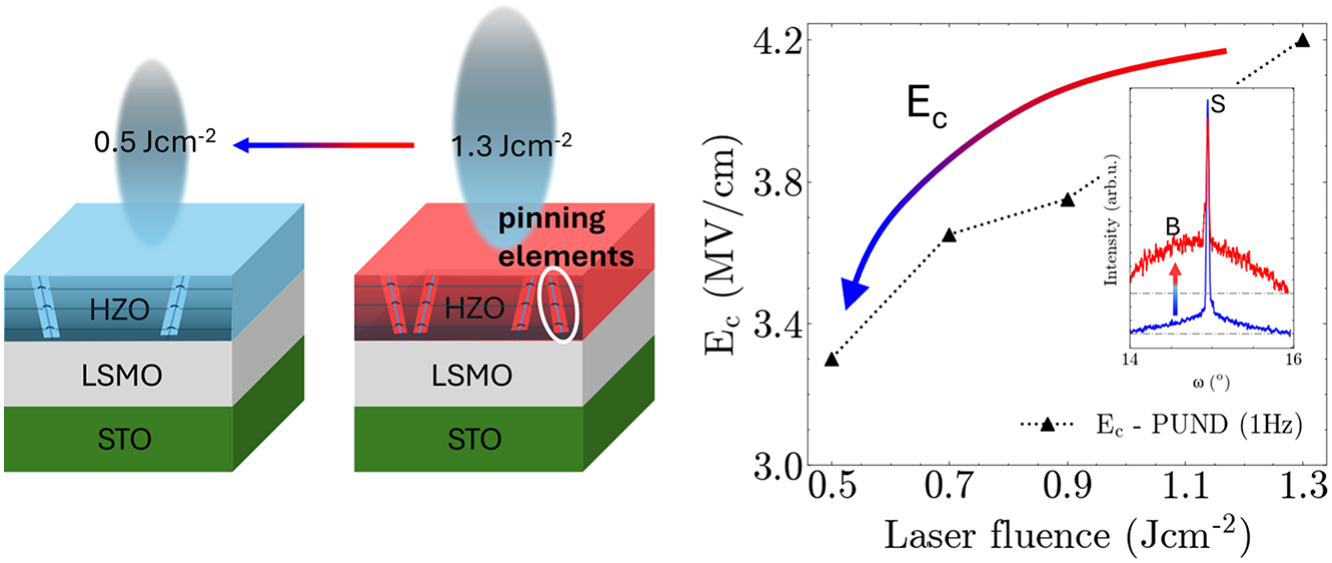

The discovery of ferroelectric hafnium oxide has spurred great interest in the semiconductor industry, enabled by its complementary metal-oxide-semiconductor compatibility and scalability. However, many questions remain regarding the origin of the ferroelectric phases and the tunability of ferroelectric properties. In this work, we explore the influence of laser fluence on coercive field (*E*_c_) in 10 nm-thick epitaxial rhombohedrally distorted orthorhombic (*r*-d *o*) Hf_0.5_Zr_0.5_O_2_ (HZO) films grown by pulsed laser deposition on La_0.7_Sr_0.3_MnO_3_-buffered (001) SrTiO_3_ substrates. When laser fluence is decreased from 1.3 J cm^–2^ to 0.5 J cm^–2^, the E_c_ decreases from ∼3.3 to ∼2.7 MV/cm. Lower laser fluence produces pure (111) oriented grains, while higher laser fluence produces an additional (11–1) orientation, leading to low angle tilt grain boundaries and associated dislocations which can act as domain pinning sites. The stabilization of the (11–1) orientation and the grain tilting at higher deposition energetics are consistent with density functional theory calculations. To achieve a low *E*_c_ in HZO, which is important for energy-efficient ferroelectric memory applications, low energetic growth conditions are required, producing the most highly perfect films.

## Introduction

In recent years, nonvolatile memory applications for energy-efficient data storage, e.g., FeRAM, have been the leading motivation for ferroelectrics research.^1^ The discovery of ferroelectricity in nanoscale films of Si-doped hafnium oxide in 2011 enthralled the ferroelectric community by offering potential for an industry-friendly CMOS compatible ferroelectric material suited for integration with Si-based electronics.^2,3^ Later, stabilization of the ferroelectricity in HfO_2_ was shown using a variety of other doping elements (Zr,^4^ La,^5^ Y,^6^ Al,^7^ etc.).

A key challenge for doped HfO_2_ is that several nonpolar polymorphs can be stabilized in the thin film form, such as the cubic (*Fm*-3*m*), the tetragonal (*P*4_2_/*nmc*), and the monoclinic (*P*2_1_/*c*) phase in HfO_2_. Therefore, it is common to find studies where the ferroelectric, orthorhombic (*o*-), or rhombohedral (*r*-) structures with space groups *Pca*2_1_^2^ or *R*3*m*,^8^ respectively, appear concomitantly with the nonpolar phases. Therefore, the control of a single-phase ferroelectric material is nontrivial but highly needed. Most previous reports on ferroelectricity in hafnia-based films consider polycrystalline multiphase films, which are only partly stabilized in the ferroelectric *o*-phase. In such films, understanding of the factors that control the ferroelectric properties is challenging. For example, studies correlating the influence of annealing on the grain size and thus ferroelectric properties also report significant differences in the phase content alongside the investigated parameter.^9,10^ By allowing for single-phase or near-single-phase stabilization, epitaxial doped-HfO_2_ films offer a simpler system for understanding the factors that control the ferroelectric properties.^11^ Such epitaxial films show no wake-up effect, which is desired for device applications.

In epitaxial films of *o*-phase doped-HfO_2_, *E*_c_ can range from ∼2 to 3 MV/cm.^12^ Here, various extrinsic factors have been shown to contribute to the phases formed and so to the ferroelectric properties, notably oxygen vacancies, dopants, strain, electric fields, and surface and interface effects.^13,14^ More recent reports identify a rhombohedrally distorted orthorhombic (*r*-d *o*) phase with polarization pointing along [001] and a unit cell elongated along *d*_111_.^15−19^ It is understood to originate from the orthorhombic phase, where it is stabilized with additional interfacial strain from the underlying substrate. On the other hand, it forms only in epitaxial films, and its measured *E*_c_ is large (∼4 MV/cm).^16,17^ Despite the demonstrated reduction of *E*_c_ with film thickness *t* as *E*_c_ ∼ *t*^–2/3^ in *o*-phase epitaxial films,^11^ the technologically relevant coercive voltage (*V*_c_) still increases with thickness as *V*_c_ ∼ *t*^1/3^, making further studies on understanding and reducing *E*_c_ (*V*_c_) critical.

In this work, we aim to understand the factor(s) that controls *E*_c_ in *r*-d *o* HfO_2_-based oxide thin films. This understanding is needed since different applications require different *E*_c_ values. We explored varying this laser fluence in films grown by pulsed laser deposition (PLD). Laser fluence influences the energetics of the deposition process, which can affect crystallinity, stoichiometry, grain orientation, defect types, and defect concentrations.^20,21^ We aim to understand which of these is the most critical controlling factor for *E*_c_.

We study epitaxial Hf_0.5_Zr_0.5_O_2_ (HZO) films grown on La_0.7_Sr_0.3_MnO_3_-buffered (LSMO) (001)-oriented SrTiO_3_ (STO) substrates at laser fluences ranging from 0.5 J cm^–2^ up to 1.3 J cm^–2^. We selected STO as the substrate and LSMO as the bottom electrode because they have been determined to yield optimal ferroelectric response in previous studies.^22,23^ We focus on the widely studied HZO composition, a solid solution of HfO_2_ and ZrO_2_ which has a fluorite-related crystal structure^24,25^ rather than aliovalent-cation-doped HfO_2_ compositions where oxygen vacancies can also form^15,26^ as these could complicate the understanding.

We find that low *E*_c_ is achieved at low laser fluence. Unlike classical perovskite ferroelectrics,^20^ where strain is increased at higher laser fluence, here we find no changes in strain but only in the films’ nanostructure with laser fluence. Films deposited at higher laser fluence show the emergence of grains with (11–1) orientation (in addition to (111) orientation, which forms at low fluence). Density functional theory (DFT) calculations are consistent with the formation of (11–1) oriented under higher energetic conditions, such as those provided by higher laser fluence. As shown both experimentally and from calculations, the additional orientation perturbs the film nanostructure, leading to grain tilting and associated dislocations, which can act as domain pinning sites to increase *E*_c_, just as in perovskite ferroelectrics.^27,28^ An *E*_c_ as low as 2.7 MV/cm was achieved for the lowest laser fluence studied, which produces the most crystallographically perfect material with a single grain orientation.

## Results

**Figure 1:**
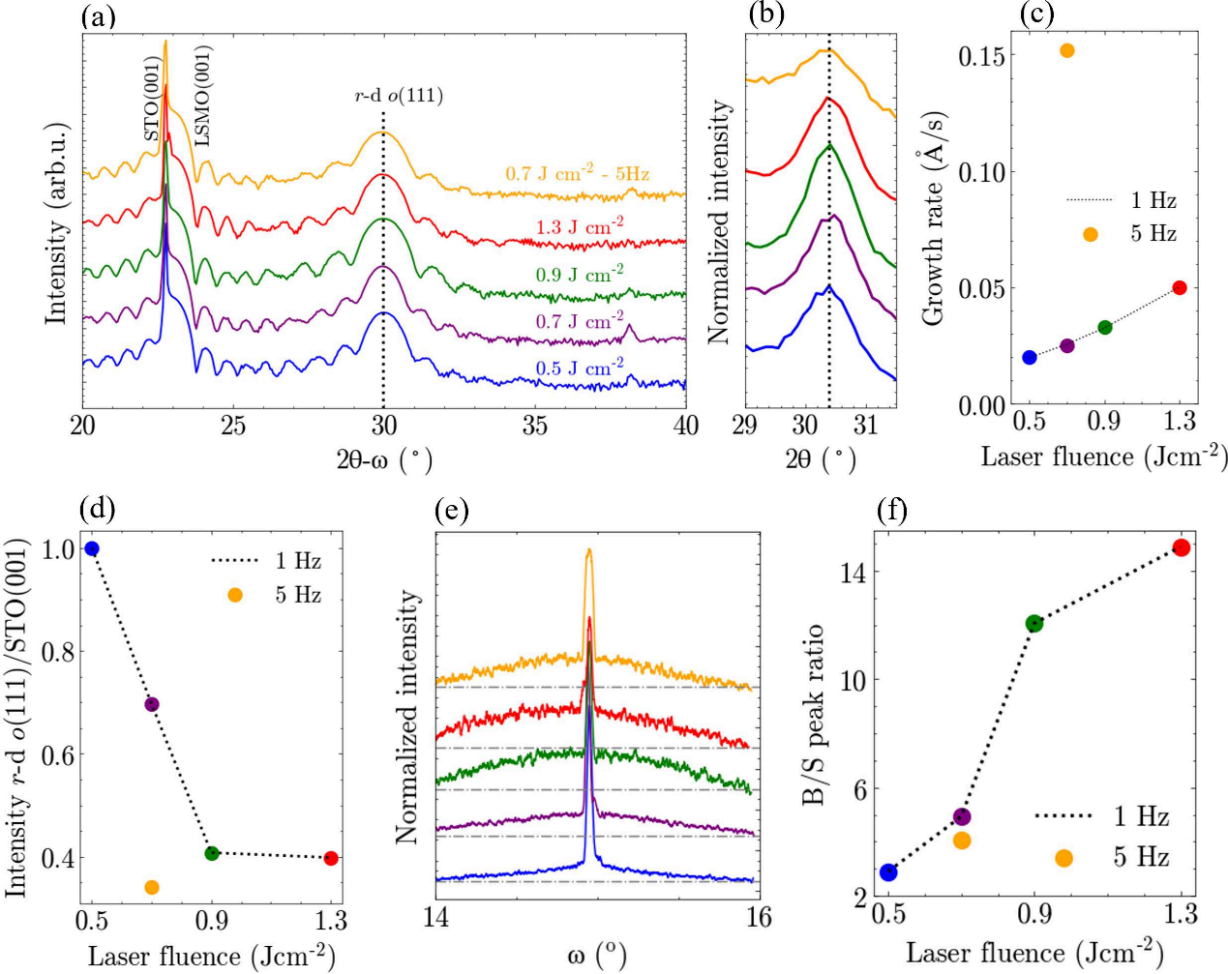
a) 2θ–ω X-ray diffraction scans of HZO films grown with different fluences. b) 2θ scans of HZO at ϕ∼45° and χ∼70°. c) Growth rate of HZO films plotted as a function of laser fluence. d) Normalized *r*-d *o*(111) peak intensity to STO (001) peak intensity as a function of laser fluence. e) Normalized ω scan (rocking curve) of HZO-0.5 (blue), 0.7 (purple), 0.9 (green), 1.3 (red), and 0.7–5 Hz (orange). Gray dotted lines indicate the baseline for broad (B) and sharp (S) peak ratio. f) B/S peak ratio as a function of laser fluence.

A series of thin film samples was deposited at a range of laser fluences, 0.5 J cm^–2^ (HZO-0.5), 0.7 J cm^–2^ (HZO-0.7), 0.9 J cm^–2^ (HZO-0.9), and 1.3 J cm^–2^ (HZO-1.3), with a laser frequency of 1 Hz. Since higher laser fluence also increases growth rate, we independently studied the influence of growth rate by depositing one film at a laser frequency of 5 Hz and a medium laser fluence of 0.7 J cm^–2^. High-resolution X-ray diffraction (XRD) scans along the 2θ–ω axes of the HZO films are shown in Figure 1a. The diffraction peak at ∼29.9° can correspond to the (111) orientation of HZO of *o-*, *r-*, or *r*-d *o*-phase HZO.^15,17,29^ We ascribe this peak to *r*-d *o*(111), for reasons discussed later. The (111) peak position remains largely unchanged across the series of samples, indicating no change in the out-of-plane lattice parameter (or strain) with laser fluence. A similar absence of strain effects from specific deposition conditions such as deposition pressure or temperature has been previously observed in epitaxial hafnia-based films.^15,30^ We propose that the absence of strain dependence on laser fluence in HZO films is due to the presence of a phase transition from the tetragonal (*t*-) to the *o*-phase during cooling and a large stiffness of polar hafnia, having about ten times larger Young’s modulus than ferroelectric perovskites.^31^ Thus, strain from the local nanostructure arising during growth is relaxed during this transition. This result is in stark contrast to previous reports on perovskite ferroelectrics, where strain levels were shown to be highly sensitive to changes in laser fluence.^20^

In Figure 1b, 2θ scans were performed at χ∼70° and ϕ∼45° to access an in-plane axis of a −111 plane. We find that all −111 peaks in all films share an equivalent 2θ position of ∼30.5°, thus implying *d*_111_ > *d*_–111_. Based on the peak positions of (111) and −111, the *d*-spacing was obtained (*d*_111_ = ∼2.99 Å and *d*_–111_ = ∼2.94 Å) using Bragg’s law. Given that the *o*-phase has α = β = γ = 90°, the rhombohedral distortion was calculated using 2 × arctan(*d*_–111_/*d*_111_), which results in α = β = γ = 89.07°,^8^ thus confirming the phase is either *r*-HZO or *r*-d *o*-HZO. To further explore the structure of the films based on the ferroelectric switching behavior, we undertook piezo-response force microscopy (PFM) measurements. We find the presence of a significant in-plane PFM response (Figure S1) and conclude that film polarization in our films is along [001]_pc_ and not [111]_pc_ and therefore that the films are stabilized in the *r*-d *o* structure.^32^

Laser fluence also changes growth rate, hence each film’s growth rate is shown in Figure 1c. With an increase in laser fluence from 0.5 J cm^–2^ to 1.3 J cm^–2^, growth rate is increased by approximately 50%. Film crystallinity is evaluated by comparing the normalized intensities of the *r*-d *o*(111) peak to the STO (001) peak in the 2θ–ω XRD scans across the sample series (see Figure 1d). We observe that film crystallinity deteriorates with an increase in laser fluence and an increase in growth rate, as would be expected. For a constant fluence of 0.7 J cm^–2^, an increase in laser frequency from 1 to 5 Hz (yellow data point) results in a more than 4-fold increase in growth rate. However, the reduction in *r*-d *o*(111) peak intensity is about the same as for the laser fluence increase from 0.5 to 1.3 J cm^–2^ where the growth rate is increased by only 50%. This indicates that there is another factor at play for the laser fluence effect beyond just degraded crystallinity with increased kinetics.

The average nanostructure of the HZO films was further analyzed by performing ω scan (rocking curves) at the (111) peak position (see Figure 1e). The rocking curves consist of a sharp peak, indicating a highly oriented crystalline region, and a broad peak, indicating the presence of some misoriented regions. Gray dotted lines indicate the baselines used for the integral breadths of the broad (B) and sharp (S) peaks, whose ratios provide a measure of grain misalignment. In Figure 1f, we plot the ratio of B and S as a function of laser fluence. For the films grown at 1 Hz laser frequency, we observe less grain misalignment for lower laser fluence. The HZO film grown with 5 Hz laser frequency shows only a slightly reduced grain misalignment than the one deposited at 1 Hz, despite the 4-fold higher growth rate than the 1 Hz sample (Figure. 1c). This is in agreement with Figure 1d, where the intensity of the *r*-d *o*(111) peak was shown to be reduced to a lesser extent than for laser energy increase. This points again to another factor at play for the laser fluence effect other than just increased kinetics for degrading grain alignment/crystallinity.

Oxygen content and cation stoichiometry are critical factors in setting ferroelectric properties in HZO films.^4,33−35^ To investigate the effect of laser fluence on film stoichiometry, we performed X-ray photoelectron spectroscopy (XPS) measurements using direct vacuum transfer (see Figure S2). We found no significant change between films grown at laser fluences of 0.5 J cm^–2^ and 1.3 J cm^–2^. This lack of stoichoimetric variation with fluence is consistent with reports on perovskite ferroelectric BaTiO_3_.^20^ The absence of composition changes with laser fluence is likely due to the similar mass, size, and the same valence of Hf and Zr ions, as well as the controlled oxygen content during the slow cooling process in high oxygen pressure (see Methods).

**Figure 2:**
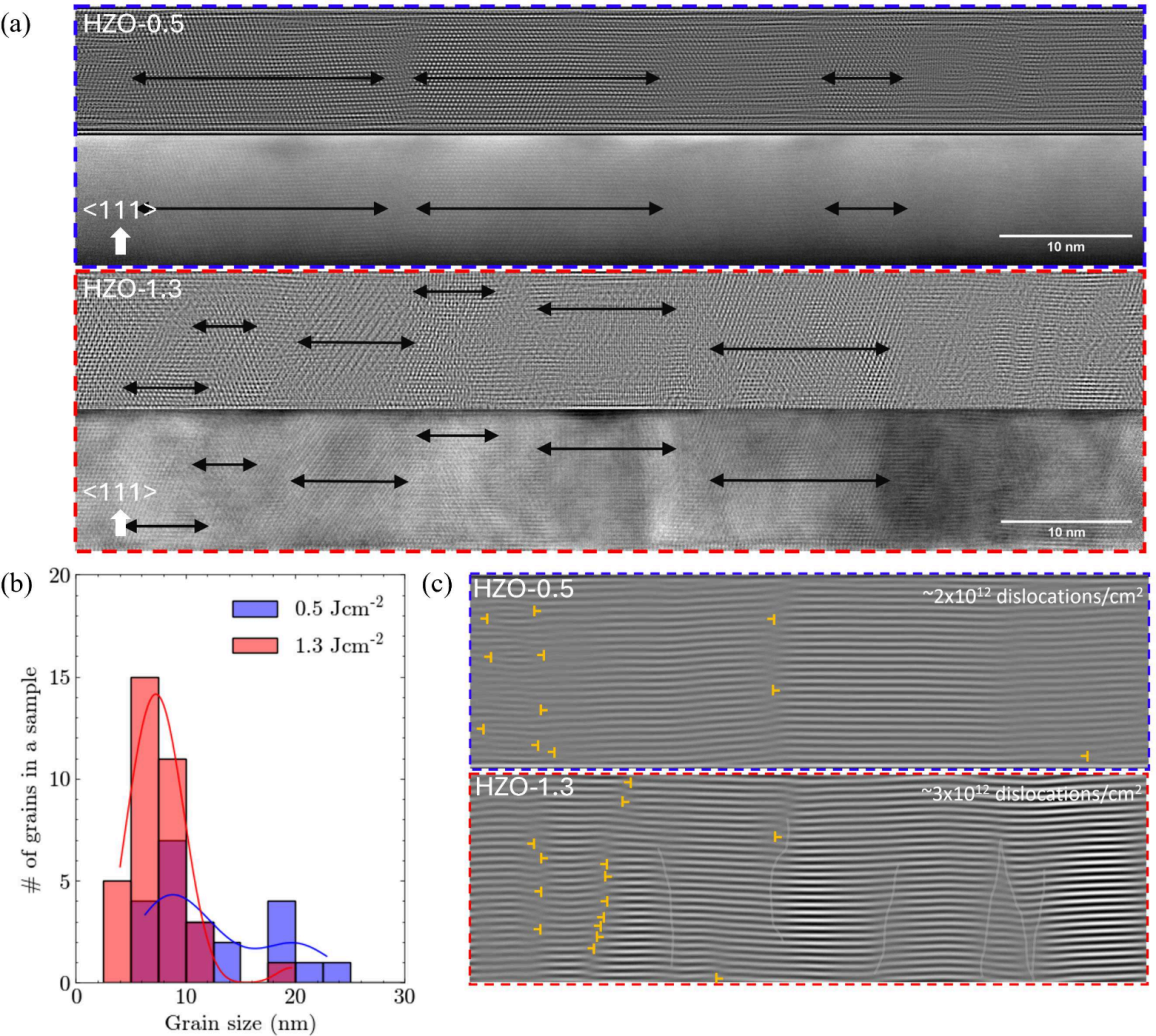
a) Fourier-filtered images (top image of each colored boxes) from cross-sectional high angle annular dark field (HAADF) images (bottom image of each colored box) of HZO-0.5 (blue outlined) and HZO-1.3 (red outlined). Zone axis is [110] STO, and ϕ = 45°. Black arrows indicate lateral grain size. b) Histogram showing grain size distribution for HZO-0.5 (blue) and HZO-1.3 (red). The average grain sizes were measured to be 12.3 and 7.7 nm, respectively, for HZO-0.5 and HZO-1.3. c) Inverse fast Fourier transform (IFFT) map of cross-sectional HAADF images (zone axis [110] STO, ϕ = 45°) of HZO-0.5 (blue) and HZO-1.3 (red). Grain tilting is visually apparent from the image. Light gray meandering vertical lines delineate discontinuities in the planes for HZO-1.3, indicative of grain boundary regions. Dislocation densities of ∼2 × 10^12^ dislocations/cm^2^ and ∼3 × 10^12^ dislocations/cm^2^ were obtained for HZO-0.5 and HZO-1.3, respectively.

We now explore the influence of laser fluence on the grain size and associated defects. Here, an extensive scanning transmission electron microscopy (STEM) analysis was performed. Based on multiple cross-sectional HAADF images (zone axis [110] STO, ϕ = 45°), the average lateral grain size was evaluated using Fourier-filtered images (Figure 2a). Fourier filtering is performed from fast Fourier transform (FFT) of HAADF images by selecting all peaks corresponding to the first order frequency of the HZO(111) lattice parameter, applying masks, and performing inverse FFT. This procedure enhances the contrast between grains, therefore allowing us to extract grain size. The HAADF images, each with a total lateral scale of 320 nm, provided sufficient data for this determination. As expected, the average lateral grain size was found to be larger, 12.3 nm, for lower laser energy film, HZO-0.5, compared to higher laser energy film, HZO-1.3, where it is 7.7 nm (Figure 2b). We further analyzed the same cross-sectional HAADF images to observe dislocations at the grain boundaries. Figure 2c shows an inverse fast Fourier transform (IFFT), visualizing horizontal dislocations. The dislocation density for HZO-0.5 is ∼2 × 10^12^ dislocations/cm^2^, while it is ∼3 × 10^12^ dislocations/cm^2^ for HZO-1.3. While this is only a broad estimate of dislocation density, it nevertheless shows the higher density of dislocations for higher laser fluence, consistent with the higher density of grain boundaries. The dislocation density values also broadly agree with those reported previously in *r*-d *o*-phase in doped-HfO_2_ work.^17^

To obtain more information about the dependence of grain tilting on laser fluence, TEM cross-section along zone axis [010] STO, ϕ = 0°, was performed to inspect interface quality between LSMO|HZO and grain boundaries. For HZO-0.5 (Figure S3a) an atomically smooth LSMO|HZO interface is observed along with a thin *t*-phase interfacial layer^8^ (inset: blue solid box). Also, minor grain tilting is observed in the body of the HZO film, consistent with the very minor B peak observed in the XRD spectrum (Figure 1e,f). The interfacial layer is tensile strained to the substrate, resulting in the in-plane lattice parameter of ∼3.91 Å, similar to the value observed in the literature.^8^ For HZO-1.3, the interface in HZO-1.3 is rougher, with an irregular thickness of a *t*-phase interfacial layer (inset: red solid box, Figure S3b). This is consistent with the more energetic particles impinging on the LSMO surface at higher laser fluence^36^ and occurs at the same time as the increased grain tilting (Figure S3b), with tilt angles of up to 2.6°, broadly consistent with the full width half-maximum of the X-ray B peak (Figure 1e,f).

**Figure 3:**
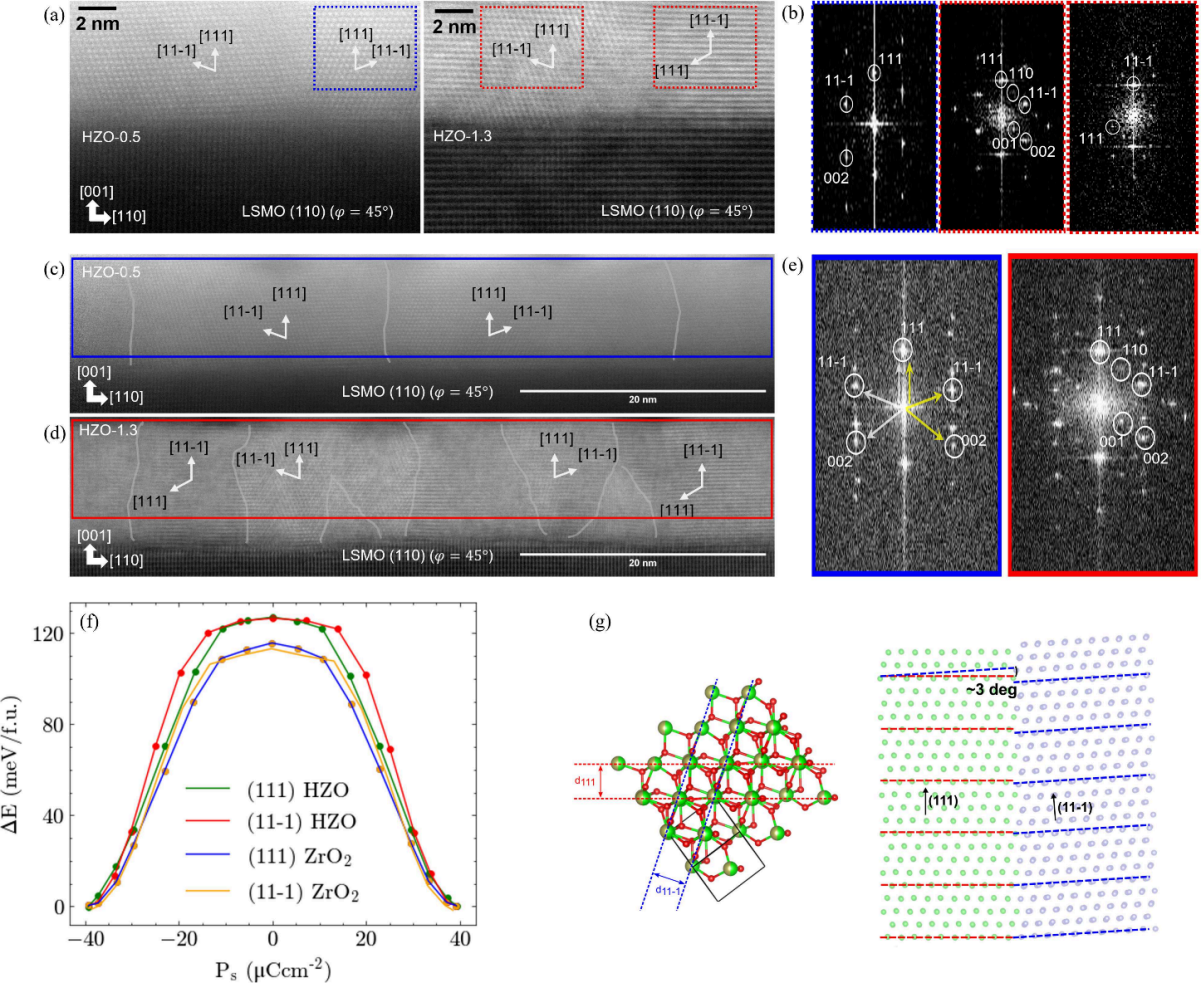
a) High-magnification cross-sectional HAADF images (zone axis [110] STO, ϕ = 45°) of HZO-0.5 and HZO-1.3. Grains are identified with their out-of-plane and in-plane orientations. b) Localized FFT was performed in different regions (blue and red dotted boxes) from HAADF images from a) for HZO-0.5 and HZO-1.3, and their out-of-plane orientation was identified based on the measured *d*-spacings. Low-magnification cross-sectional HAADF images (zone axis [110] STO, ϕ = 45°) of c) HZO-0.5 and d) HZO-1.3. (111) and (11–1) grain orientations were observed from HZO-1.3, but only a single (111) orientation was observed from HZO-0.5. Grain boundaries were identified in gray. e) FFTs were performed across the whole HAADF images from c,d) to sample multiple regions with different orientations (HZO-0.5: blue and HZO-1.3: red). f) Polarization switching barrier for the out-of-plane component of the spontaneous polarization P_s_ for the (111) and (11–1) oriented *r*-d *o*-phase of ZrO_2_ and HZO. g) An atomistic representation of the *r*-d *o*-HZO unit cell, highlighting the [111] and [11–1] directions. On the right, a schematic representation of the tilted grains between (111) (green atoms) and (11–1) (mauve atoms) orientation is shown. The *x* direction is aligned with the in-plane direction in the (111) oriented grain. Only cations are shown for the sake of clarity.

Having shown that higher laser fluence yields smaller grains, more grain tilting, and associated dislocations, we now explore how laser fluence influences grain alignment. To do this, we further investigated cross-sectional HAADF iamges of HZO-0.5 and HZO-1.3, now along the zone axis [110] STO, ϕ = 45°. High-magnification images and their FFT images are shown in Figure 3a,b and lower-magnification images and their FFT images in Figure 3c–e.

The grain orientations were identified from *d*-spacing measurements from the localized FFTs as shown in Figure 3b. FFTs from HZO-0.5 (blue box from Figure 3b) and HZO-1.3 (middle red box from Figure 3b) give an out-of-plane *d*-spacing of 2.99 Å and in-plane *d*-spacing of 2.93 Å, which agrees with XRD results from Figure 1a,b. On the other hand, the far right red dotted boxed FFT from Figure 3b demonstrates an out-of-plane *d*-spacing of 2.93 Å and in-plane *d*-spacing of 2.99 Å, which is indicative of a (11–1)-oriented grain. Therefore, we assign both (111) and (11–1) grain orientations for HZO-1.3, while only a single (111)-oriented *r*-d *o*-grain is assigned for HZO-0.5. The absence of a distinct 2θ peak related to (11–1)-oriented grains in XRD is due to several factors: the lower fraction of (11–1) relative to the (111) grains, the overlap of expected peaks from the two orientations, the lower structure factor of (11–1) compared to (111), the tilt of the (11–1) grains out-of-plane relative to the (001) direction of the substrate, and the increased mosaicity of the (11–1)-oriented grains (Figure S4). To provide more statistical information about the grain sizes and orientations in the two samples, low-magnification images of the two samples were obtained (Figure 3c,d) with FFTs shown in Figure 3e. It is immediately apparent that smaller grains are present for HZO-1.3 (Figure 3d) compared to HZO-0.5 (Figure 3c), also in agreement with the grain size analysis from Figure 2a,b. Also, the grain orientations (Figure 3e) are consistent with those measured at higher magnification (Figure 3b).

Next, DFT calculations were undertaken to understand the relative stability of the (111) and (11–1) orientations. Calculations were performed on the *r*-d *o*-phase where α = β = γ = 89.07°, from which the (111) and (11–1) oriented HZO cells were obtained. The surface energy (*E*_s_) for the *r*-d *o*-phase showed *E*_s(111)_ = 1.08 J m^–2^ and *E*_s(11–1)_ = 1.15 Jm^–2^, indicating that the (11–1) oriented surface is slightly less favored than the (111) oriented surface, and so the (111) oriented grains are more stable (Figure 3f). Under higher energetic conditions, such as high laser fluence (HZO-1.3 film), the presence of the additional (11–1) orientation is enabled. To understand whether the presence of the (11–1) oriented grains impacst the ferroelectric properties, the energy barrier and polarization were calculated along the ferroelectric switching paths of (111) and (11–1) oriented HZO grains. No significant differences in either P_s_ or barrier height were found indicating that *E*_c_ would not be increased simply because of switching of the (11–1) domains.

The calculated interplanar distances for the (111) and (11–1) orientations align well with the HRTEM and XRD results, yielding *d*_111_ = 2.997 Å and *d*_11–1_ = 2.934 Å. The detailed structural parameters of the (111) and (11–1) oriented HZO cells can be found in Table S1. Because of the slight differences in the lattice vectors and angles between the (111) and (11–1) orientations, the tilting between the boundaries and associated dislocations is expected at the (111)/(11–1) grain boundary (Figure 3g). Indeed, the DFT calculations predicted a grain tilt of about 3°, which broadly agrees with the experimental observations (Figure 1e,f, Figure S3b). The formation of the additional (11–1) grains which are intrinsically stabilized at higher laser energy is consistent with the finer grain size of HZO-1.3 compared to the HZO-0.5 (Figures 2b and 3c,d).

Finally, on the microstructural side, energy dispersive spectroscopy (EDS) was performed based on the HAADF images (Figures S5–S6). A homogeneous distribution of cations and sharp interfaces was observed in both HZO-0.5 and HZO-1.3 films, corroborating the wide-range XPS results.

**Figure 4:**
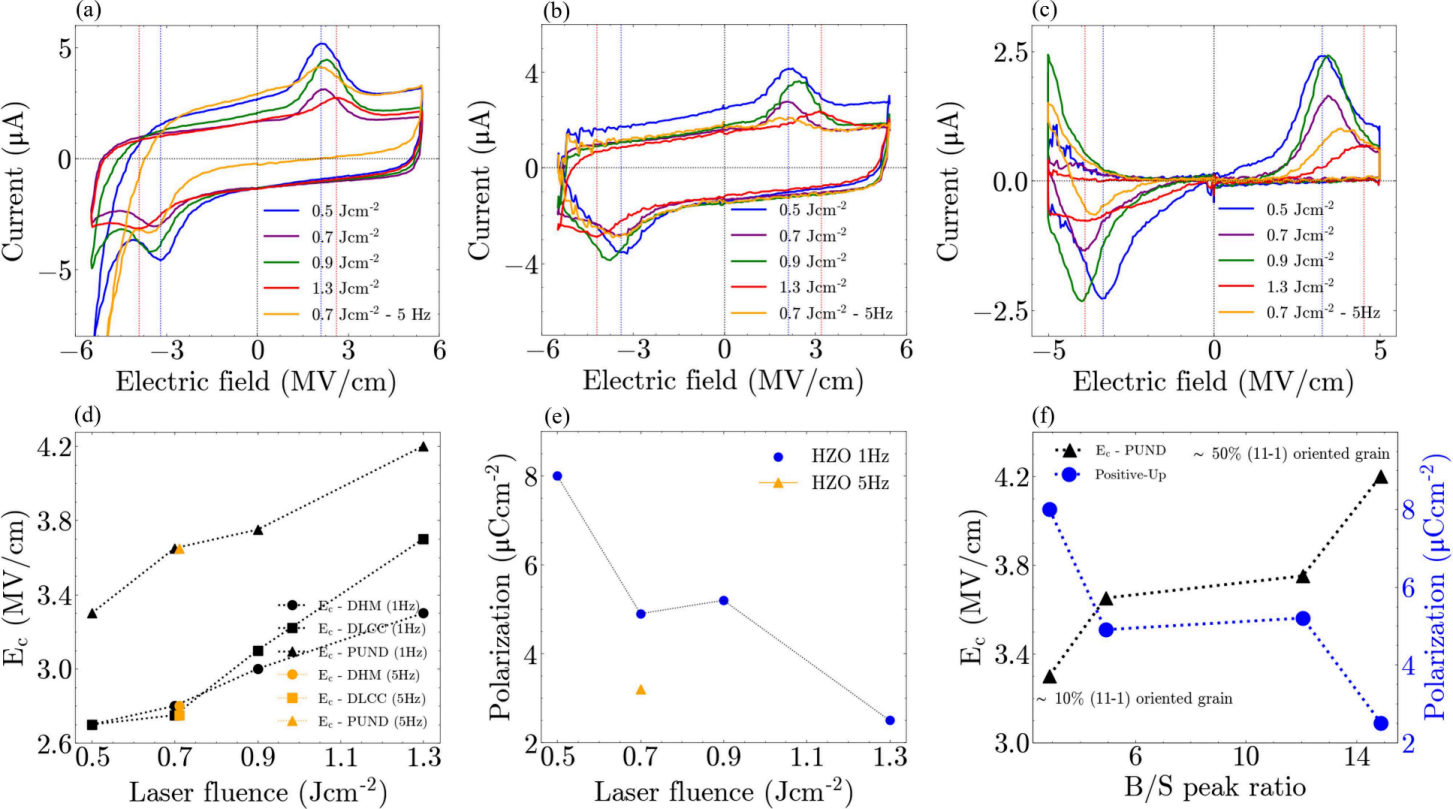
Current–electric-field loops for HZO films from a) dynamic hysteresis measurement (DHM), b) dynamic leakage current compensation (DLCC), and c) positive-up–negative-down (PUND) measurement. Vertical blue and red lines indicate ferroelectric switching peaks for HZO-0.5 and HZO-1.3. d) Dependence of *E*_c_ on laser fluence based on different measurement protocols. e) Dependence of polarization value on laser fluence based on positive-up (PU) measurement. f) Dependence of *E*_c_ (from PUND) and polarization (from PU) on the B/S peak ratio.

We now investigate the influence of laser fluence on the ferroelectric properties of HZO films on a macroscopic scale. For electrical measurements, 50-μm diameter electrodes were patterned using UV lithography, and tungsten (W) was sputtered as the top electrode. Figure 4a–c shows the current–electric-field (*I*–*E*) relation measured by different protocols: dynamic hysteresis measurement (DHM), dynamic leakage current compensation (DLCC), and positive-up–negative-down (PUND). DHM shows all current contributions, including the dielectric, ferroelectric, and leakage currents. DLCC corrects for the leakage current following a built-in software function.^37^ PUND includes only the ferroelectric contribution. All films show wake-up-free ferroelectric behavior. All HZO films demonstrate clear ferroelectric switching peaks at both negative and positive bias (see Figure. 4a–c). The coercive peak values are extracted from the current maxima positions to avoid the influence of leakage. The loops are off-centered by ∼−0.5 MV/cm because of the asymmetric LSMO|HZO|W device stack.^38^ The *E*_c_ was calculated as the average of fields of current maxima on the positive and negative bias. Corresponding polarization–electric field (*P*–*E*) and *I*–*E* loops for all of the HZO samples with different measurement protocols can be found in Figures S7–S10.

Figure 4d displays *E*_c_ as a function of laser fluence. *E*_c_ was observed to increase from ∼2.7 to ∼3.3 MV/cm with an increase in laser fluence from 0.5 J cm^–2^ to 1.3 J cm^–2^ from DHM. The trend of increasing *E*_c_ with laser fluence is consistent across all three measurement protocols. Notably, the sample grown at 5 Hz exhibits the same *E*_c_ value as the one deposited at 1 Hz with the same laser fluence of 0.7 J cm^–2^, despite its poorer crystallinity (overall level of defects in the sample, Figure 1d). This indicates that factors other than overall crystalline perfection influence *E*_c_.

Figure 4e shows the dependence of *P*_r_ on laser fluence based on positive-up (PU) measurements. PU measurements were chosen due to variations in leakage current at negative bias (see Figures S8–S9). The higher leakage current in films deposited at lower fluence might arise from their higher polarization value leading to more accumulation of bound charges and enhanced carrier injection. HZO-1.3 demonstrates much lower *P*_r_ (<3 μCcm^–2^) compared to HZO-0.5 (∼8 μCcm^–2^), at 5 MV/cm. The marked reduction in *P*_r_ for the 5 Hz deposition rate (∼3 μC cm^–2^) is consistent with the strong reduction in crystallinity (Figure 1d). We recall 5 Hz increases the deposition rate by 4-fold (Figure 1c). We also recall that 5 Hz does not produce more grain tilting (Figure 1e,f and Figure S3a) and so microstructure plays less of a role in controlling *P*_r_ than random defects do. Figure 4f shows the dependence of *E*_c_ and *P*_r_ (from PU measurement) on the B/S peak ratio. A trend of higher *E*_c_ and reduced *P*_r_ is observed with higher B/S ratio, i.e., more grain tilting.

Overall, we have shown that high laser fluence leads to increased *E*_c_. This occurs when two grain orientations, (111) and (11–1), are stabilized. At the same time, no change in strain (Figure 1a,b and Figure 3d), composition (Figure S2), or switching barrier height (Figure 3f) takes place. We also showed that *E*_c_ is not reduced by defects associated with the higher growth rate of 5 Hz (Figure 4d). Thus, we can deduce that the increased *E*_c_ with laser fluence is dominated by the grain tilting effect of the (111)/(11–1) grain boundaries (Figure 4f), along with associated grain boundary dislocations (Figure 2c). While the grain boundary defects also lead to a decrease in *P*_r_, the defects associated with the higher growth rate appear more significant for controlling *P*_r_ (as seen from the 5 Hz point in Figure 4e). Overall, *E*_c_ is controlled by domain wall pinning by dislocations associated with tilted grains, similar to the case of perovskite ferroelectrics.^39−41^

While *o*-phase films can exhibit higher *P*_r_ values,^11^ similar values of *P*_r_ (sub-10 μCcm^–2^) were achieved in *r*-d *o*-phase epitaxial hafnia-based films of similar thickness,^17^ but the *E*_c_ values observed previously are usually higher than 4 MV/cm.^15,17^ However, in this work, in films of ∼10 nm thickness, by tuning the laser energy, we demonstrated a further reduction in *E*_c_ (by ∼30%) to sub-3 MV/cm (∼2.7 MV/cm). Tuning *E*_c_ at a specific thickness is an important step toward the integration of ferroelectrics in memory and logic devices for next generation electronics.^42,43^

## Conclusion

In epitaxial rhombohedrally distorted orthorhombic (*r*-d *o*) Hf_0.5_Zr_0.5_O_2_ (HZO) films where composition, strain, and thickness are kept constant, we have demonstrated a link between nanostructure and coercive field (*E*_c_). We showed that increasing laser fluence leads to increased nanostructural disorder via the emergence of a (11–1) grain orientation, which emerges in addition to the (111) orientation, which is present at low laser fluence. The (11–1) orientation is shown to produce in-plane grain tilting and dislocations at the low angle grain boundaries, and these imperfections are consistent with increased domain pinning and increased *E*_c_. Low laser fluence, which produces a higher proportion of (111) orientation and high crystallinity, leads to a roughly 30% reduction in *E*_c_ compared to literature values on the *r*-d *o*-phase (to ∼ 2.7 MV/cm at 0.5 J cm^–2^). Overall, our work shows the importance of controlling nanostructure to tune the ferroelectric properties of HZO films, enabling performance optimization of hafnia for memory devices.

## Methods

### Deposition of Thin Films

To make the HZO target, HfO_2_ and ZrO_2_ powders were ground and mixed for an hour and compressed into a pellet which was sintered for 8 h at 1400 °C. The LSMO target was fabricated with LaCO_3_, SrCO_3_, and MnO powders, which were first calcined at 850 °C and then sintered at 1200 °C. Epitaxial 10 nm-thick films of Hf_0.5_Zr_0.5_O_2_ (HZO) were grown with a range of fluences ((0.5, 0.7, 0.9, 1.3) J cm^–2^) on LSMO-buffered (37 unit cells) TiO_2_-terminated STO substrates via PLD using a KrF excimer laser with a wavelength of 248 nm. LSMO buffer layers were grown at 750 °C under 100 mTorr (0.133 mbar) oxygen partial pressure with a laser fluence of 0.7 J cm^–2^ and a laser frequency of 2 Hz. The number of unit cells for LSMO was tracked by using reflection high energy electron diffraction (RHEED). HZO films were grown with a range of laser fluence under a 75 mTorr (0.1 mbar) oxygen atmosphere at 890 °C and a laser frequency of 1 Hz, unless stated otherwise. The spot size was kept constant at 2.5 mm^2^ for all the depositions with a target to substrate distance of 5 cm. After deposition, the heterostructures were cooled to room temperature at a rate of 5 °C min^–1^ under 0.4 bar of oxygen partial pressure.

### Characterization of Thin Films

A PANalytical Empyrean Diffractometer was used for XRD characterization. STEM was conducted using a Thermo Fisher Scientific Spectra 300. Energy dispersive spectroscopy was performed to consolidate constituents in each layer. For XPS characterization, the films were transferred in situ from the PLD chamber to an attached XPS analysis chamber. An Al K_α_1 X-ray source and a SPECS PHOIBOS 150 hemispherical analyzer were used to collect high resolution O-1s, Zr-3d, and Hf-4f core-level spectra. For electrical measurements, 50 μm-diameter electrodes were patterned using a UV lithography mask. Top electrodes were sputtered using DC sputtering. Lift off was done with acetone. Characterization of ferroelectricity was conducted using AixACCT TF analyzer 2000.

### DFT Calculations

DFT calculations were performed using VASP 6.4.1,^44−47^ and the GGA approximation was used along with Perdew–Burke–Ernzerhof functional for solids (PBEsol).^48^ The plane-wave cutoff energy was set to 600 eV, while the size of the Γ-centered K-Point mesh was fixed to 4 × 4 × 1 grid for bulk HZO calculations, 4 × 4 × 3 grid for ZrO_2_ bulk calculations, and 4 × 4 × 1 grid for ZrO_2_ slabs, for which a vacuum of 15 Å was introduced as well. HZO *r*-phase unit cells were constructed with alternating Hf and Zr layers along the *c*-axis.

Structural relaxations for initial and final structures were carried on until forces converged to a threshold of 1 meV/Å. Surface energies have been calculated from 5-layer and 6-layer ZrO_2_ slabs using the Boettger^49^ approach. ZrO_2_ was used in place of HZO for surface energy calculations, as the HZO surface could not be well-defined. Switching energy barriers were calculated using the climbing image nudged elastic band (CI-NEB) method, as implemented in the VTST routines of Henkel group,^50^ with a force convergence threshold of 10 meV/Å and using 9 images per calculation. The spontaneous polarization was calculated using the modern theory of polarization, as implemented in VASP.^51,52^ The atomistic representations were generated using VESTA.^53^

## Data Availability

The data that support the findings of this study are available from the corresponding authors upon request.

## Supporting Information Available

Further details on the relative stoichiometric and homogeneous distribution of cations using X-ray photoelectron spectroscopy and electron–electron dispersive spectroscopy; detailed structural properties of rhombohedrally distorted orthorhombic phase of ZrO_2_ and HZO; detailed explanation on ferroelectric measurement protocols; supporting figures (PDF)
