# Supplementary material for: Coercive Field Control in Epitaxial Ferroelectric Hf0.5Zr0.5O2 Thin Films by Nanostructure Engineering
Source: ACS Appl Mater Interfaces. 2025 Apr 15;17(17):25442–50. doi: 10.1021/acsami.4c21787 (PMC12051166; doi:10.1021/acsami.4c21787)
Supplement: Supplementary file 1 — am4c21787_si_001.pdf [file am4c21787_si_001.pdf]

Supporting Information: Coercive field control in epitaxial ferroelectric $Hf_{0.5}Zr_{0.5}O_2$ thin films by nanostructure engineering

Ji Soo Kim[1,⋆], Nives Strkalj[1, †], Alexandre Silva[2,3], Veniero Lenzi[2,4], Luis Marques[2,3], Megan O. Hill[1, ‡], Ziyi Yuan[1], Yi-Xuan Liu[1], Maximilian T. Becker[1], Simon M. Fairclough[1], Caterina Ducati[1], Yizhi Zhang[5], Jianan Shen[5], Zedong Hu[6], Hongyi Dou[5], Haiyan Wang[5,6], José P. B. Silva[2,3,⋆], and Judith L. MacManus-Driscoll[1,⋆]

[1] *Department of Materials Science & Metallurgy, University of Cambridge, 27 Charles Babbage Road, Cambridge CB3 0FS, United Kingdom*

[2] *Physics Center of Minho and Porto Universities (CF-UM-UP), University of Minho, Campus de Gualtar, 4710-057 Braga, Portugal*

[3] *Laboratory of Physics for Materials and Emergent Technologies, LapMET, University of Minho, 4710-057 Braga, Portugal*

[4] *CICECO – Aveiro Institute of Materials, Department of Chemistry, University of Aveiro, 3810-193 Aveiro, Portugal*

[5] *School of Materials Engineering, Purdue University, West Lafayette, IN 47907, USA*

[6] *Elmore Family School of Electrical and Computer Engineering, Purdue University, West Lafayette, IN 47907, USA*

[†] *Present address: Center for Advanced Laser Techniques, Institute of Physics, 10000 Zagreb, Croatia*

[‡] *MAX IV Laboratory and Department of Physics, Lund University, 22 100 Lund, Sweden*

[⋆] *Correspondence should be sent to: Ji Soo Kim (jsk55@cam.ac.uk) or Judith L. MacManus-Driscoll (jld35@cam.ac.uk) or José P. B. Silva (josesilva@fisica.uminho.pt)*

Structural Charaterization

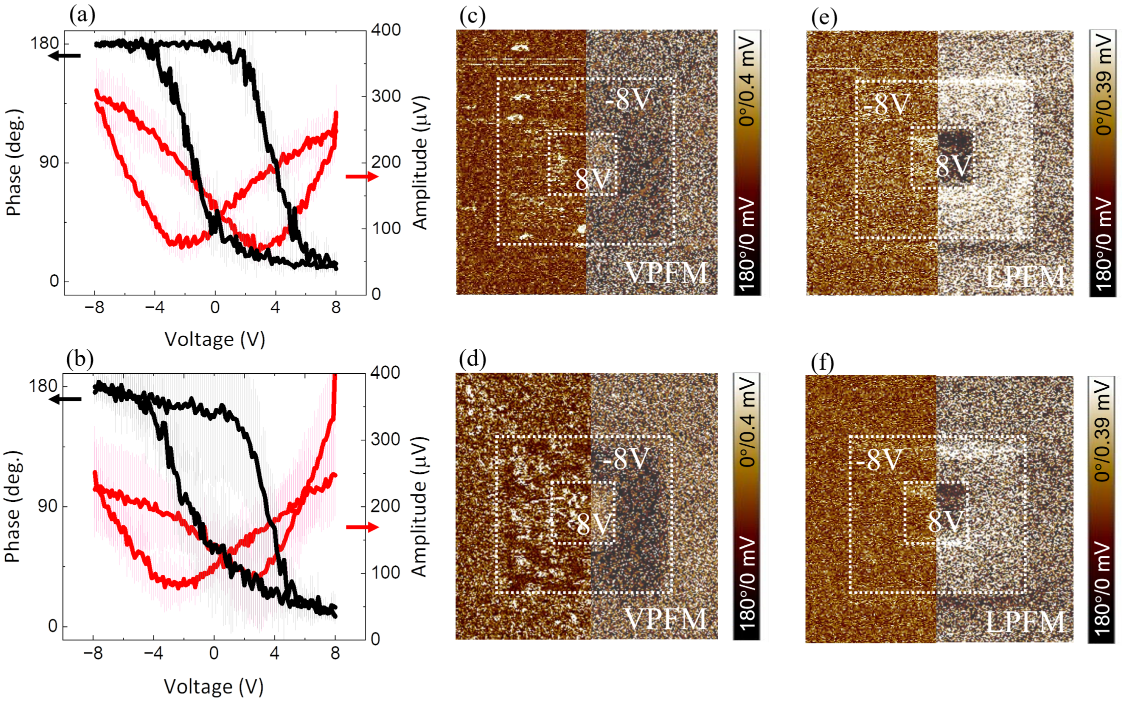

Figure S 1: Averaged piezo-electric force spectroscopy (PFS) out-of-plane (OOP) hysteresis loops in phase (black) and amplitude (red) of a) HZO-0.5 and b) HZO-1.3 with V_{ac} of 1V at 10 kHz. Piezo-electric force microscopy (PFM) signals with square oppositely polarized domains with -8V and 8V from HZO-0.5 and HZO-1.3. OOP phase contrast and amplitude response of c) HZO-0.5 and d) HZO-1.3 shown with vertical-PFM (VPFM). In-plane (IP) phase contrast and amplitude repsonse of e) HZO-0.5 and f) HZO-1.3 shown with lateral-PFM (LPFM).

Piezoelectric force microscopy (PFM) was performed on HZO-0.5 and HZO-1.3 with Pt coated tip. Initially, piezo-electric force spectroscopy (PFS) was performed with V_{dc} (writing voltage) of -8 and 8V with V_{ac} (reading voltage) of 1V at 10kHz. Average coercive voltages were estimated based on amplitude minima, and are 2.5 V for HZO-0.5 and 2.9 V for HZO-1.3 respectively. Based on the PFS response, -8 and 8 V were applied in order to ensure full saturation of polarization for vertical-PFM (VPFM). Figure.S1c-d show VPFM signals with clear contrast for out-of-plane (OOP) polarization with 180° of phase change for HZO-0.5 and HZO-1.3. They demonstrate clear bi-stable states that can be polarized with given field. We confirmed that pristine state includes multiple domains which consists of both up and down polarized states. VPFM also show amplitude signals. They demonstrate constant amplitude across the polarized region with clear domain wall between two oppositely polarized domain. Polarization was switched using -8

and 8V in order to ensure fully saturated polarization. Figure.S1e-f show lateral-PFM (LPFM) signals for in-plane (IP) polarization with 180° of phase and amplitude change for HZO-0.5 and HZO-1.3. They show constant amplitude across the polarized region with clear domain walls between two oppositely polarized domains.

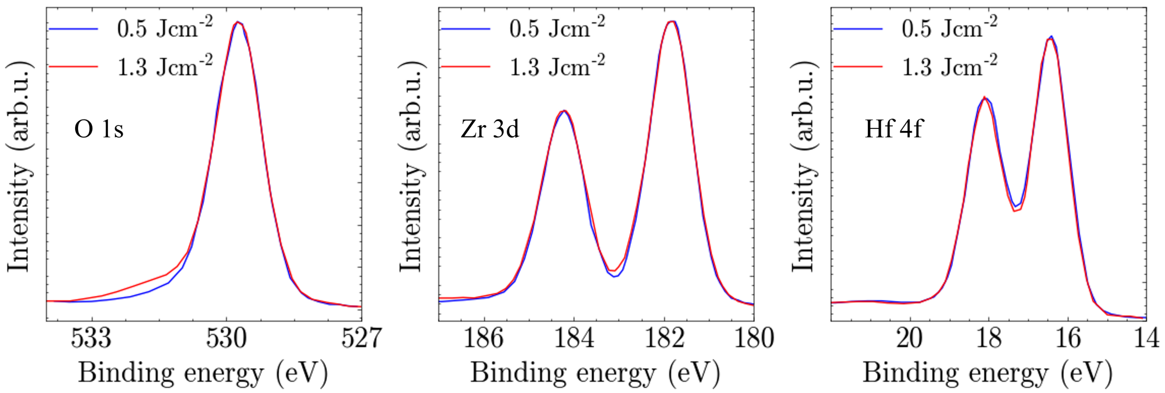

Figure S 2: In-situ x-ray photoelectron spectroscopy (XPS) of Hf 4f, Zr 3d and O 1s peaks on HZO-0.5 and HZO-1.3.

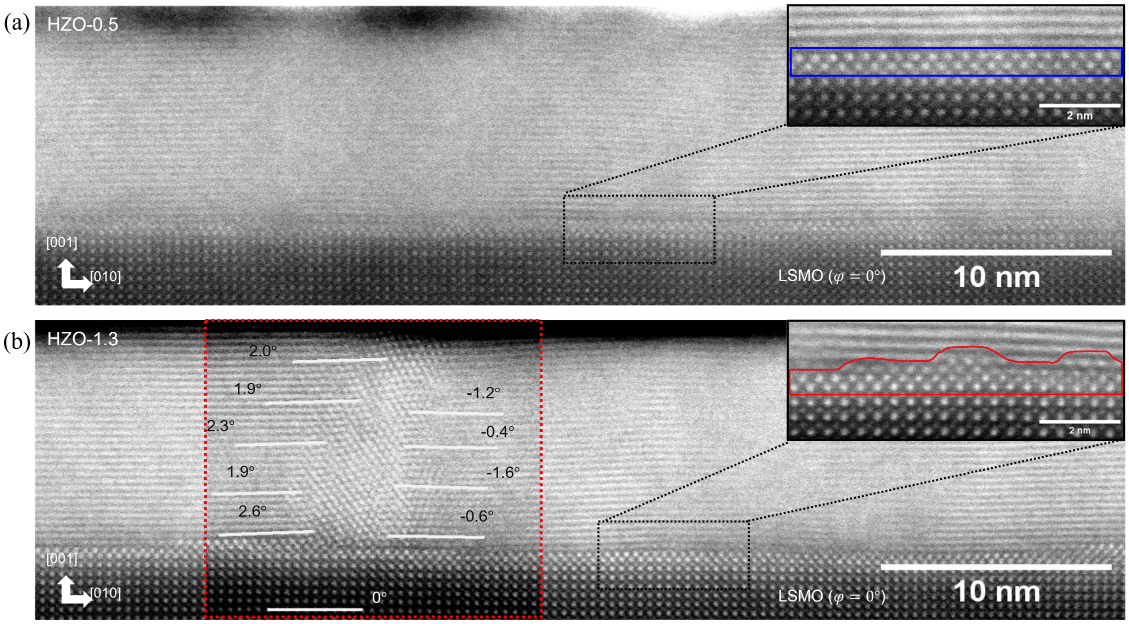

Figure S 3: Cross-sectional high angle annular dark field (HAADF) images of a) HZO-0.5 and b) HZO-1.3 along [010] zone axis ($\phi = 0°$) for grain size and interfacial layer analysis

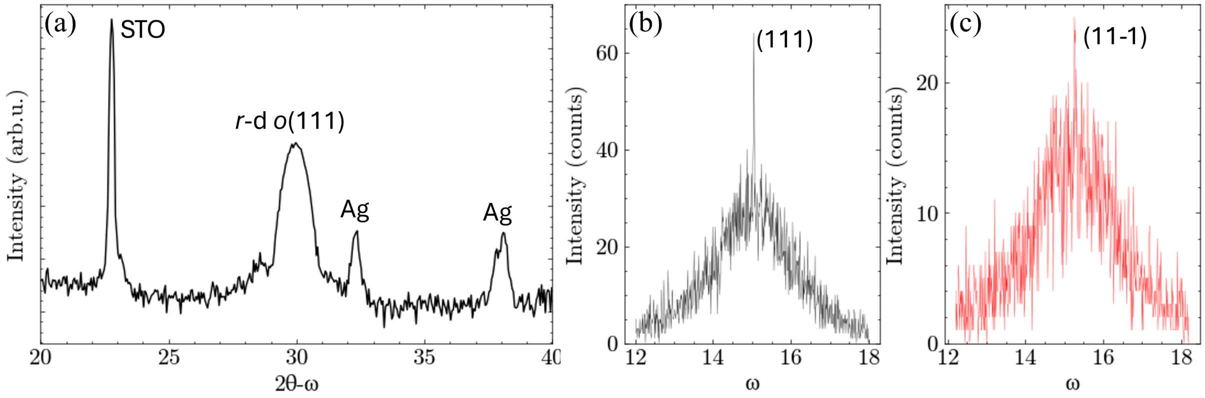

Figure S 4: a) 2θ-ω aligned to (11-1) crystallite at 2θ ∼ 30.4°. ω scan (rocking curve) of b) (111) and c) (11-1). Presence of (11-1) peak at ω ∼ 15.27° was observed

	Orientation	a (Å)	b (Å)	c (Å)	α (deg.)	β (deg.)	γ (deg.)	d (Å)	P_s (μCcm^{-2})	ΔU (meV/f.u.)
ZrO_2	111	7.2423	7.2287	9.0206	91.88	88.30	121.26	3.0069	39.2341	108.9
	11-1	7.3444	7.2399	8.8280	91.71	87.38	120.74	2.9427	38.0382	108.7
HZO	111	7.2195	7.2060	17.9844	91.88	88.30	121.26	2.9974	39.5726	122.0
	11-1	7.2059	7.3375	17.6055	92.44	88.11	120.75	2.9342	39.3821	121.8

Table S 1: Structural properties of (111) and (11-1) oriented rhombohedrally distorted orthorhombic (r-d o) phase of ZrO_2 and HZO. The out-of-plane distance d is also reported, along with the polarization and the polarization switching barrier ΔU.

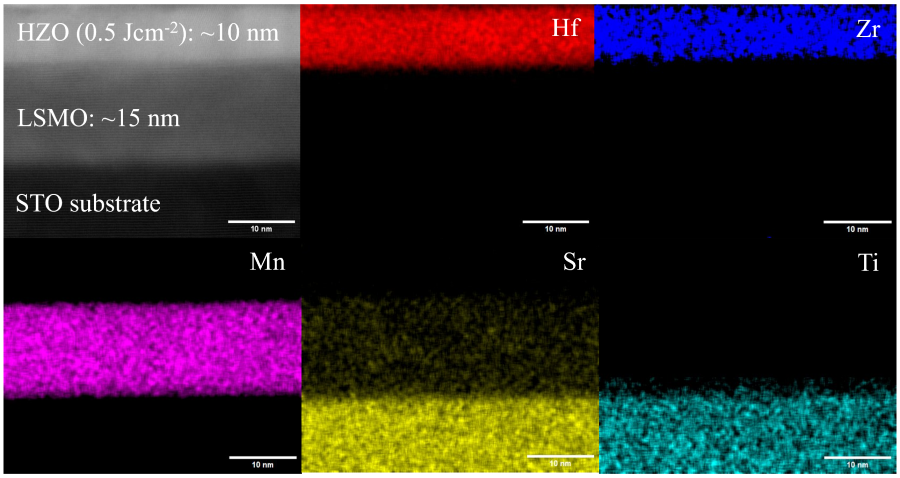

Figure S 5: Cross-sectional HAADF image of HZO- 0.5 for EDS analysis. Each layer's thickness was identified where HZO and LSMO are ∼10 nm and 15 nm respectively. EDS mapping of Hf (top center) and Zr (top right) in HZO, Mn (bottom left) and Sr (bottom center) in LSMO, and Sr and Ti (bottom right) in STO. Homogenous distribution of Hf and Zr ions is found within HZO. No significant inter-diffusion is detected across STO|LSMO and LSMO|HZO interfaces.

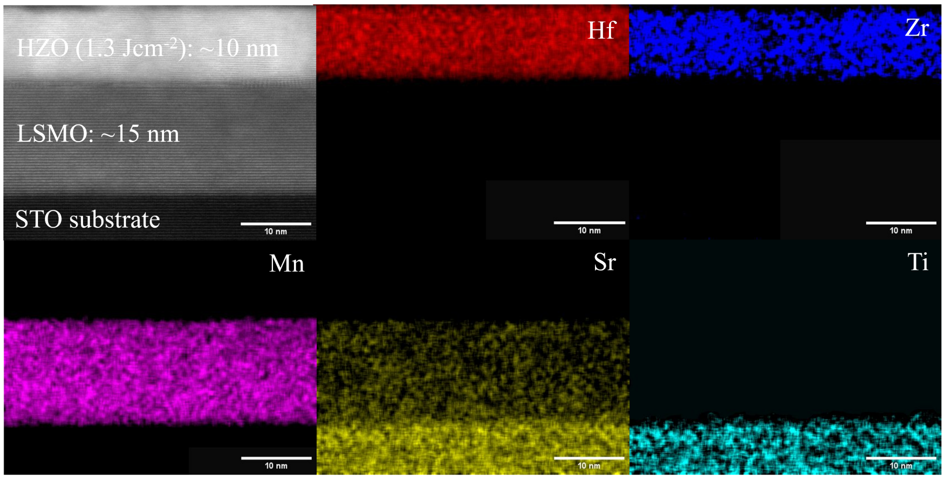

Figure S 6: Cross-sectional HAADF image of HZO-1.3 for EDS analysis. Each layer's thickness was identified where HZO and LSMO are ∼10 nm and 15 nm respectively. EDS mapping of Hf (top center) and Zr (top right) in HZO, Mn (bottom left) and Sr (bottom center) in LSMO, and Sr and Ti (bottom right) in STO. Homogenous distribution of Hf and Zr ions is found within HZO. No significant inter-diffusion is detected across STO|LSMO and LSMO|HZO interfaces.

Ferroelectric measurement

Polarization-electric field (P-E) loops are obtained by integrating the current-voltage loops for dynamic hysteresis measurement (DHM) and dynamic leakage current compensation (DLCC) protocols. All HZO films show polarization saturation P_s with the increase in electric field, a common characteristic of ferroelectrics, and do not show a wake-up effect. DLCC measurements yield a remanent polarization P_r without leakage contributions allowing a comparison among films deposited at different fluences.

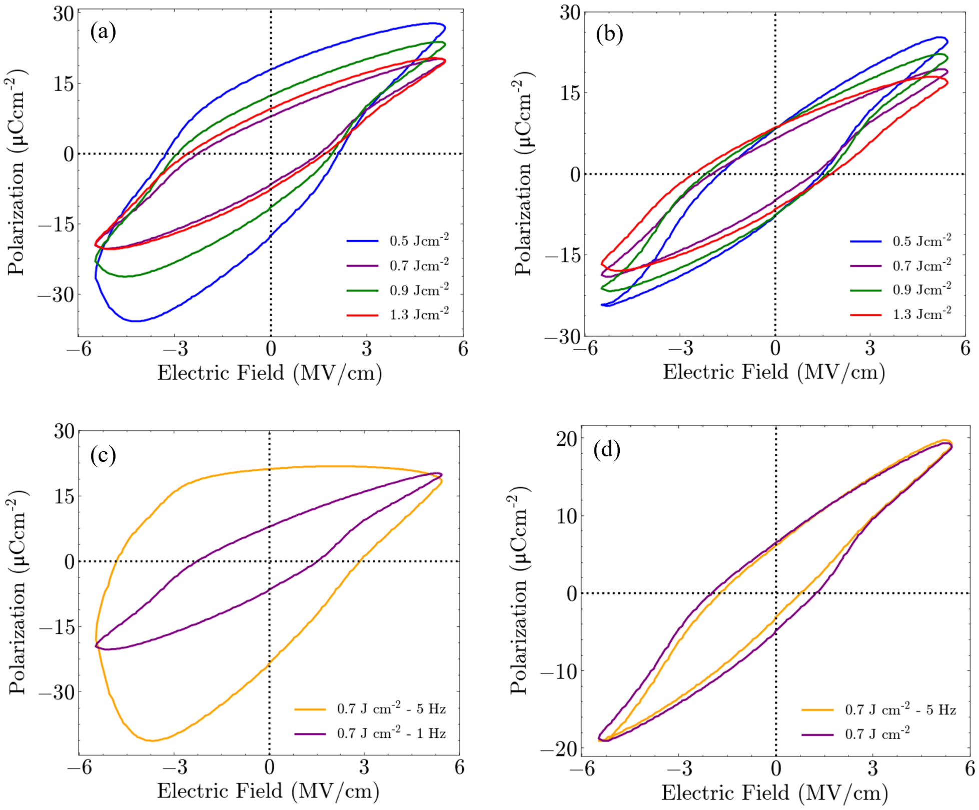

Figure S 7: P-E loops of HZO films grown with 0.5, 0.7, 0.9 and 1.3 Jcm^{-2} from a) dynamic hysteresis measurement (DHM) and b) dynamic leakage current compensation (DLCC) measurement. P-E loops for HZO-0.7 grown with two different rates, 1 Hz (purple) and 5 Hz (orange) from c) DHM and d) DLCC measurement

We note that most integrated devices operate over a high frequency range (MHz $\sim$ GHz) where conduction currents become negligible. Thus, DLCC measurements allow visualization of typical P-E loops in actual devices [1].

Figure.S7a-b show polarization-electric field relation for HZO films grown at 0.5 Jcm^{-2} (HZO-0.5), 0.7 Jcm^{-2}

(HZO-0.7), 0.9 Jcm^{-2} (HZO-0.9), and 1.3 Jcm^{-2} (HZO-1.3) from DHM and DLCC. Figure.S 7c-d show polarization-electric field relation for HZO films grown at 0.7 Jcm^{-2} at 1 Hz and 5 Hz from DHM and DLCC. They all demonstrate saturation of polarization with increase in electric field with P_s. Although all the films are ferroelectric in nature without doubt, they demonstrate different level of leakage current, therefore, their P_r values based on DHM is not reflecting directly about the HZO film.

Positive-up-negative-down (PUND) measurement

Positive-up (PU) measurements were done in order to get an insight about sample's true P_r or $2P_r$ value. They were performed with three voltage pulses, first pulse with -5 MV/cm for pre-poling, and second and third pulses with 5 MV/cm. The current response from second and third pulses were then subtracted, resulting in polarization displacement current only.

Polarization evaluation based on PUND is not significantly affected by conduction current (resistance) or its change with polarization reversal for the P and U pulses.

1) The current-field (I-E) data presented in Figure.S8 show overall low leakage currents at positive bias used for polarization evaluation and thus negligible contribution in P_r.

2) The P-E loops in Figure.S10 in show saturation and a flat polarization with field upon field removal as expected for ideal ferroelectric loop without other contributions. Thus, no significant change of conduction current occurred with polarization switching.

3) The ratio of current at coercive field to current at zero field is more than a factor of ten for films deposited at 0.5 Jcm^{-2} and more than hundred for films deposited at other fluences, showing that current tends to zero at zero field, as expected in the absence of resistive switching. The higher amount of current at zero field for films deposited at 0.5 Jcm^{-2} could be coming from the tail of the ferroelectric switching peak which is at lower coercive field than in other films.

Polarization-Electric field (P-E) loops for HZO films grown with 0.5, 0.7, 0.9 and 1.3 Jcm^{-2} from a) dynamic hysteresis measurement (DHM) and b) dynamic leakage current compensation (DLCC) measurement. Polarization-Electric field (P-E) loops for HZO-0.7 grown with two different rates, 1 (purple) and 5 Hz (orange) from b), dynamic hysteresis measurement (DHM) and c) dynamic leakage current compensation (DLCC) measurement.

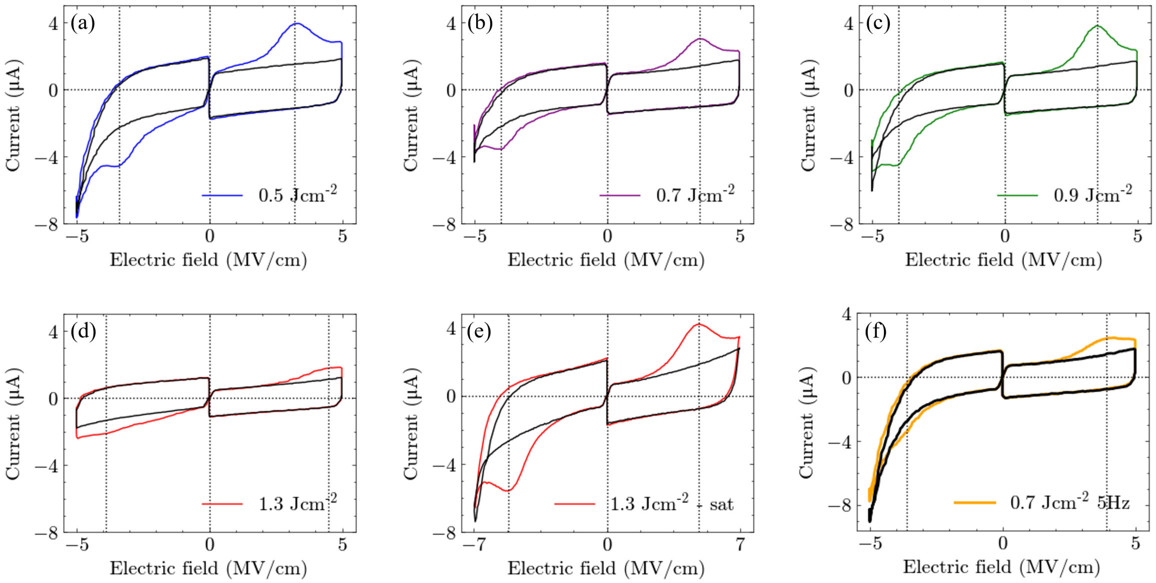

Figure S 8: a-d) I-E response from PUND measurement with positive bias for HZO-0.5, 0.7, 0.9 and 1.3 with 5 MV/cm. e) I-E response from PUND measurement for HZO-1.3 with 7 MV/cm for its polarization to saturate. f) I-E response from PUND measurement for HZO-0.7 grown with 5 Hz of laser pulse frequency.

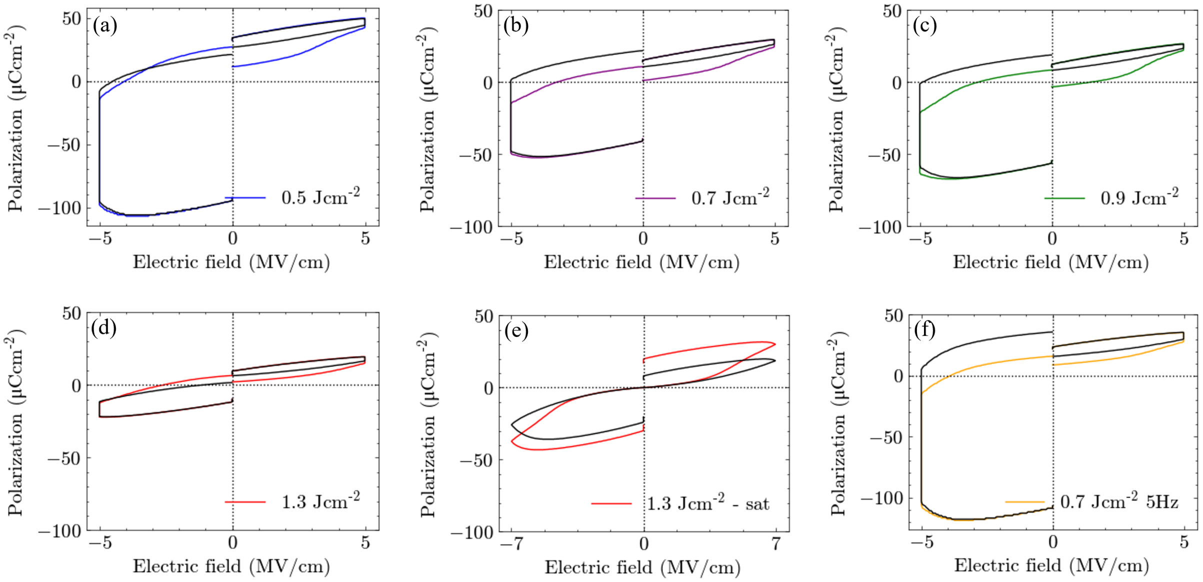

Figure S 9: a-d) P-E response from PUND measurement with positive bias for HZO-0.5, 0.7, 0.9 and 1.3 with 5 MV/cm. e) P-E response from PUND measurement for HZO-1.3 with 7 MV/cm for its polarization to saturate. f) P-E response from PUND measurement for HZO-0.7 grown with 5 Hz of laser pulse frequency.

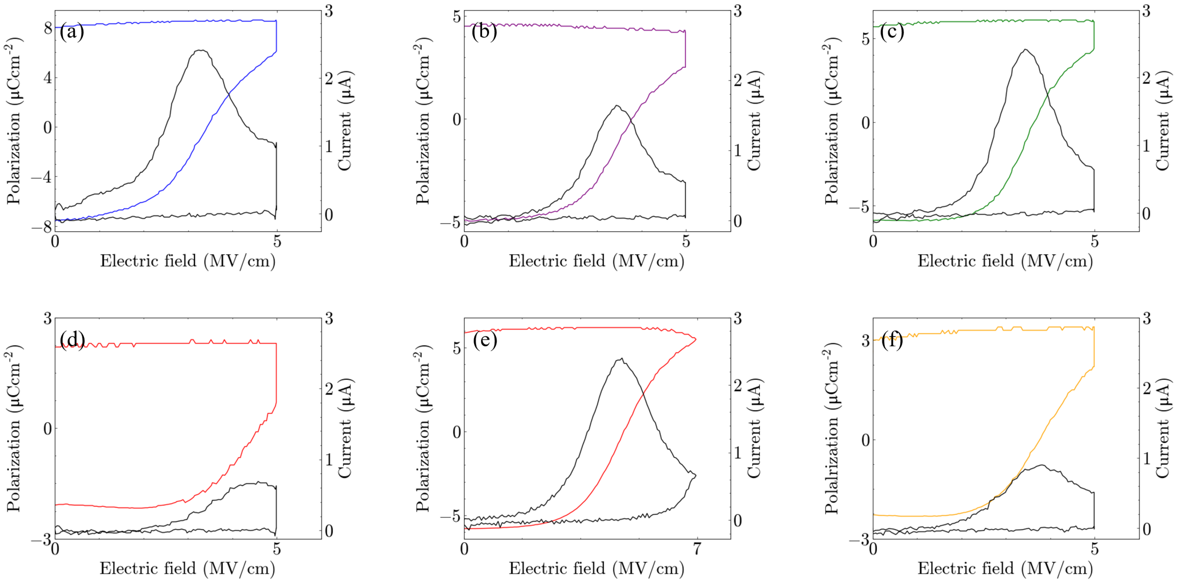

Figure S 10: a-d) I-E and P-E responses from PU measurement with positive bias for HZO-0.5, 0.7, 0.9 and 1.3 with 5 MV/cm. e) I-E and P-E responses from PU measurement for HZO-1.3 with 7 MV/cm for its polarization to saturate. f) I-E and P-E responses from PU measurement for HZO-0.7 grown with 5 Hz of laser pulse frequency.

References

(1) Meyer, R.; Waser, R.; Prume, K.; Schmitz, T.; Tiedke, S. Dynamic leakage current compensation in ferroelectric thin-film capacitor structures. *Applied Physics Letters* **2005**, *86*.
